# Systemic inflammation and changes in physical well-being in patients with breast cancer: a longitudinal study in community oncology settings

**DOI:** 10.1093/oncolo/oyae212

**Published:** 2024-08-23

**Authors:** Nikesha Gilmore, Yue Li, Christopher L Seplaki, Michael Sohn, Ying Yang, Chin-Shang Li, Kah Poh Loh, Po-Ju Lin, Amber Kleckner, Mostafa Mohamed, Paula Vertino, Luke Peppone, Karen Mustian, Sindhuja Kadambi, Steven W Corso, Benjamin Esparaz, Jeffrey K Giguere, Supriya Mohile, Michelle C Janelsins

**Affiliations:** Department of Surgery, Division of Supportive Care in Cancer, University of Rochester Medical Center, Rochester, NY 14642, United States; Department of Public Health Sciences, University of Rochester Medical Center, Rochester, NY 14642, United States; Department of Public Health Sciences, University of Rochester Medical Center, Rochester, NY 14642, United States; Department of Psychiatry, University of Rochester Medical Center, Rochester, NY 14642, United States; Department of Biostatistics and Computational Biology, University of Rochester Medical Center, Rochester, NY 14642, United States; Department of Public Health Sciences, University of Rochester Medical Center, Rochester, NY 14642, United States; Department of Surgery, Division of Supportive Care in Cancer, University of Rochester Medical Center, Rochester, NY 14642, United States; Division of Hematology/Oncology, Department of Medicine, Wilmot Cancer Institute, University of Rochester Medical Center, Rochester, NY 14642, United States; Department of Surgery, Division of Supportive Care in Cancer, University of Rochester Medical Center, Rochester, NY 14642, United States; Department of Pain and Translational Symptom Science, University of Maryland School of Nursing, Baltimore, MD 21201, United States; Department of Public Health Sciences, University of Rochester Medical Center, Rochester, NY 14642, United States; Division of Hematology/Oncology, Department of Medicine, Wilmot Cancer Institute, University of Rochester Medical Center, Rochester, NY 14642, United States; Department of Biomedical Genetics, University of Rochester Medical Center, Rochester, NY 14642, United States; Department of Surgery, Division of Supportive Care in Cancer, University of Rochester Medical Center, Rochester, NY 14642, United States; Department of Surgery, Division of Supportive Care in Cancer, University of Rochester Medical Center, Rochester, NY 14642, United States; Division of Hematology/Oncology, Department of Medicine, Wilmot Cancer Institute, University of Rochester Medical Center, Rochester, NY 14642, United States; Upstate Carolina NCI Community Oncology Research Program, Spartanburg, SC 29303, United States; Heartland NCI Community Oncology Research Program, Decatur, IL 62526, United States; NCI Community Oncology Research Program of the Carolinas, Greenville, SC 29615, United States; Division of Hematology/Oncology, Department of Medicine, Wilmot Cancer Institute, University of Rochester Medical Center, Rochester, NY 14642, United States; Department of Surgery, Division of Supportive Care in Cancer, University of Rochester Medical Center, Rochester, NY 14642, United States

**Keywords:** physical well-being, resilience, systemic inflammation, patients with breast cancer, chemotherapy

## Abstract

**Background:**

Chemotherapy adversely affects physical well-being and inflammation may be related to changes in physical well-being. We evaluated the association of systemic inflammation with changes in physical well-being.

**Methods:**

In a prospective study of 580 patients with stages I-III breast cancer we assessed immune cell counts, neutrophil:lymphocyte ratio (NLR), lymphocyte:monocyte ratio (LMR), and platelet:lymphocyte ratio (PLR) within 7 days before chemotherapy (pre-chemotherapy). Physical well-being was assessed using the Functional Assessment of Cancer Therapy: General—Physical Well-being subscale (FACT-PWB) pre-chemotherapy and 1 month and 6 months post-chemotherapy. Clinically meaningful decline in physical well-being was determined as decreasing FACT-PWB by more than one point from pre-chemotherapy level, and non-resilience defined as having decline post-chemotherapy and not returning to within one-point of pre-chemotherapy FACT-PWB by 6 months post-chemotherapy. Multivariable logistic regressions examined the association between inflammation and changes in physical well-being, adjusting for sociodemographic and clinical characteristics.

**Results:**

Fifty-nine percent (310/529) and 36% (178/501) of participants had physical well-being decline post-chemotherapy and 6 months post-chemotherapy, respectively. Fifty percent (147/294) were non-resilient. Low NLR and PLR were associated with 1.78 (*P* = .01) and 1.66 (*P* = .02) fold greater odds of having a decline in physical well-being 6 months post-chemotherapy compared to those with high NLR and PLR, respectively. Low NLR and PLR were associated with 1.92 (*P* = .02) and 2.09 (*P* = 0.01) fold greater odds of being non-resilient 6 months post-chemotherapy compared to those with high NLR and PLR, respectively.

**Conclusion:**

Low NLR and PLR were associated with chemotherapy-induced changes in physical well-being independent of sociodemographic and clinical risk factors.

## Teaser

Chemotherapy adversely affects physical well-being, and inflammation may be related to changes in physical well-being. This article evaluates the association of systemic inflammation with changes in physical well-being.

**Implications for practice**

In patients with breast cancer receiving chemotherapy, low levels of NLR and PLR prior to the start of treatment are associated with a decline in physical well-being and non-resilience up to 6 months after completing treatment. These profiles may guide treatment discussions by identifying patients who are at increased risk chemotherapy-induced decline in physical well-being and reduced ability to regain pre-treatment levels of physical well-being.

## Background

Patients with cancer exhibit a high prevalence of symptoms including pain, nausea, and fatigue, which are strongly correlated with diminished physical function.^1^ The increased burden of these symptoms can adversely affect both physical functionality and overall quality of life.^2,3^ Furthermore, patients with cancer receiving chemotherapy are at increased risk of chronic decline in physical well-being, an important component of health-related quality of life, that is closely associated with physical functioning.^4,5^ Decline in physical well-being can lead to loss of independence, which is a devastating consequence for survivors of cancer.^6^ As the number of survivors of cancer continues to rise, it is imperative to determine which patients with breast cancer are at increased risk of decline in physical well-being after treatment to guide the development of physical well-being interventions for cancer survivors. In addition to enhancing the physical well-being of patients with cancer, these interventions may also improve functional outcomes for survivors, given the established association between physical well-being and functional status.^4,5,7^

Patients with breast cancer receiving chemotherapy can exhibit heterogeneous outcomes of physical well-being after treatment.^8,9^ Some patients will be *resistant to decline* in measures of physical well-being and complete treatment without any significant adverse effects on physical well-being throughout cancer therapy.^10^ Some will be “*resilient,” returning to their pre-chemotherapy level of physical well-being, and some will be* “*non-resilient,” not regaining their baseline physical well-being.*^10^

Inflammatory markers are associated with changes in physical function.^4,11^ Given the connection between physical function and physical well-being, blood-based inflammatory biomarkers might also be possible candidates for markers of changes to physical well-being. Systemic inflammation can be indicated by a change in the relative numbers of different leukocyte subsets and platelets. While there are more precise ways to measure systemic inflammation (eg, Interleukin-6) these measures are expensive and not typically part of routine clinical practice. A more easily obtained measure of systemic inflammation is the total and differential counts of neutrophils, lymphocytes, monocytes, neutrophil to lymphocyte ratio (NLR), lymphocyte to monocyte ratio (LMR), and platelet to lymphocyte ratio (PLR)^12,13^

In patients with cancer, inflammatory markers are associated with frailty,^14^ mortality,^15-17^ and poor prognosis.^18^ However, the relationship between systemic markers of inflammation with chemotherapy-induced changes in physical well-being in patients with breast cancer has not yet been described. We leveraged an existing multicenter prospective cohort to investigate whether pre-chemotherapy inflammatory markers are associated with decline in physical well-being, resistance to decline, and non-resilience after completing chemotherapy. We hypothesized that patients with pre-chemotherapy increased systemic inflammation would be more likely to have decline in physical well-being and be at greater risk of being non-resilient. Previous research has assessed resilience primarily through measures centered on functional quality of life. This research enhances the existing literature by utilizing the Functional Assessment of Cancer Therapy (FACT) General—Physical Well-Being (PWB) subscale, which incorporates the interplay between symptoms and functional status, to assess changes in physical well-being and the recovery of physical well-being after the completion of chemotherapy.

## Methods

### Study design, settings, and participants

We conducted a secondary analysis of data from a large nationwide prospective cohort study of 580 patients with breast cancer (ClinicalTrials.gov identifier NCT01382082) that enrolled participants with stages I-III breast cancer and scheduled to begin a course of adjuvant or neo-adjuvant chemotherapy and paired non-cancer controls from 2011 to 2013.^19,20^ Patients were recruited throughout the US from the University of Rochester Cancer Center (URCC) National Cancer Institute (NCI) Community Oncology Research Program (NCORP) Community Affiliates. Measures were completed before the first cycle of chemotherapy (pre-chemotherapy), within 4 weeks after the last chemotherapy cycle (1-month post-chemotherapy), and 6 months after the last chemotherapy cycle. For this study, we included all patients with breast cancer who had data available for total and differential leukocytes from clinical labs as previously described.^21^ Institutional review boards at the URCC NCORP Research Base and at each of the NCORP Community Affiliates approved the study. All participants provided informed consent before completing study requirements.

### Primary exposures: immune cell composition

Laboratory data taken from the medical record were obtained pre-chemotherapy; mean = 12.2 (SD = 17.8) days before the first day of chemotherapy.^21^ Total white blood cells (WBC), neutrophils, lymphocytes, monocytes, and platelets were reported in 10^3^ cells/µL and extracted from the medical records by study staff. A hematology and geriatric medicine board certified physician (RM) reviewed all lab values to confirm reporting accuracy and discrepancies were clarified according to standard operating procedures. NLR, LMR, and PLR were calculated. Neutrophils, lymphocytes, monocytes, NLR, LMR, PLR, and total WBC were dichotomized at the median (low = below the median, high = equal to or above the median). To date there are no established clinically meaningful cutoffs for neutrophils, lymphocytes, monocytes, NLR, LMR, PLR, and total WBC, thus we chose cutoffs at the median for each inflammatory marker, consistent with previous work.^22^

### Outcome variables: measures of physical well-being

Physical well-being was assessed using the FACT-PWB subscale. The FACT-PWB was completed pre-chemotherapy, 1 month, and 6 months post-chemotherapy and contains 7-items with a score ranging from 0 to 28 with lower scores indicating lower physical well-being.

Decline in physical well-being was measured from pre-chemotherapy to post-chemotherapy and pre-chemotherapy to 6 months post-chemotherapy. Clinically meaningful decline in physical well-being was defined as a greater than one-point decrease in the FACT-PWB score from pre-chemotherapy according to guidelines for longitudinal changes specifically in FACT-PWB scores.^23^

Resilience and resistance in physical well-being were measured 6 months post-chemotherapy. Given that there are no established measures of resilience and resistance using the FACT-PWB, we used the same guidelines for longitudinal changes in FACT-PWB scores.^23^ Patients were considered non-resilient if they had a decline in physical well-being 1-month post-chemotherapy and did not return to within one point of their pre-chemotherapy FACT-PWB score by 6 months post-chemotherapy. Patients were considered resistant if they did not have a decline in physical well-being 1-month post-chemotherapy and had a FACT-PWB score within one point of their pre-chemotherapy score by 6 months post-chemotherapy.

### Covariates

Covariates include patient demographics: age, race, education, cancer stage, menopausal status, and marital status. We included the number of days between the pre-chemotherapy blood draw and the start of chemotherapy as a covariate in the models because this time window could influence immune cell counts.^24^ Furthermore, since the pre-chemotherapy FACT-PWB score influences the 1 month and 6 months post-chemotherapy FACT-PWB score, the pre-chemotherapy FACT-PWB score was included as a covariate. Sociodemographic factors (eg, education, race, income, marital status) are associated with both changes in immune cell composition and physical well-being.^21,25,26^

Participants self-reported age, race, education, and marital status. Menopausal status was categorized into pre-menopausal, post-menopausal, peri-menopausal, and medically induced. Other demographic variables were dichotomized accordingly: race (white vs non-white), education (high school or below vs some college or above), and marital status (married/long-term relationship vs other). Cancer stage (II and III vs I), treatment type (adjuvant vs neoadjuvant), type of chemotherapy (anthracycline vs non-anthracycline), body mass index, date that the complete blood count was obtained, and the start date of chemotherapy were extracted from the medical records.

### Statistical analyses

Descriptive statistics were used to summarize the baseline characteristics of the sample as well as the proportion of the sample with decline in physical well-being. All analyses were conducted using complete-case data because it offers a more transparent and reliable approach in this context, considering the nature of the predictors. This method avoids the additional assumptions and potential biases that may arise from imputing biomarker values. A “univariable” generalized estimating equations (GEE) model^27^ with logit link and an exchangeable structure were used to assess the associations between baseline level of an inflammatory marker at pre-chemotherapy (baseline inflammatory marker) and physical well-being declines post-chemotherapy and 6 months post-chemotherapy. The model included time treated as nominal, baseline inflammatory marker, and time-by-baseline inflammatory marker interaction.

A multivariable GEE model with logit link and an exchangeable structure was used to assess the associations between baseline inflammatory marker and physical well-being declines post-chemotherapy and 6 months post-chemotherapy. The model included time treated as nominal, baseline inflammatory marker, time-by-baseline inflammatory marker interaction, baseline physical well-being, age, race (white vs other), education (some college or above vs other), marital status (married/long term vs other), menopause status (peri vs pre, post vs pre, and medical induce vs pre), cancer stage (II vs I, III vs I, and IV vs I), body mass index, treatment type (adjuvant vs neo-adjuvant), growth factor (yes vs no), and time from lab blood draw to start of chemotherapy.

Separate bivariate and multivariable logistic regression analyses were then used to assess the associations between baseline levels of inflammatory markers being non-resilient 6-months post-chemotherapy (o*nly patients with physical well-being decline from pre-to post-chemotherapy were included in this analysis);* being resistant 6-months post-chemotherapy (only patients who did not have physical well-being decline from pre-to post-chemotherapy were included in this analysis). In sensitivity analyses, each biomarker was categorized into quartiles. We examined the associations between the highest quartile of biomarkers (reference group: the highest quartile) with the aforementioned 4 outcomes.^28^

Analyses were performed using either SAS v. 9.4 (SAS Institute) and JMP Pro v. 15 (SAS Institute). Sankey diagrams were used to visualize the longitudinal changes to patients’ physical well-being from the start of chemotherapy to 6 months after the completion of chemotherapy. The Sankey diagrams were made using SankeyMATIC http://sankeymatic.com. All tests were 2-tailed and *P* < .05 was used to assess statistical significance.

## Results

### Demographic and clinical characteristics

A summary of participants’ baseline sociodemographic and clinical characteristics is shown in Table 1. All female patients (*n* = 580) with breast cancer from the parent study were included in the analysis.^19-21^ Patients had mean age of 53.4 years [range: 22-81; SD: 10.6 years). Patients’ mean pre-chemotherapy inflammatory markers are shown in Table 1. The median cut point for the pre-chemotherapy inflammatory markers are as follows: total WBC = 7.0 (range = 1.0-27.4), lymphocytes = 1.8 (range = 0.0-15.7), monocytes = 0.5 (range = 0.0-8.9, neutrophils = 4.1 (range = 0.0-22.7), platelets = 257.5 (range = 91.0-735.0), NLR = 2.27 (range = 0.1-39.5), LMR = 3.75 (0.0-694.0), and PLR = 145.27 (range = 21.4-1560.0).

**Table 1.** Baseline sociodemographic and clinical characteristics. Table 1 presents the baseline sociodemographic and clinical characteristics of study participants. The columns represent the entire study population, the group that experienced a decline in physical well-being post-chemotherapy, and the group that experienced a decline in physical well-being at the 6 months post-chemotherapy timepoint. The rows list various characteristics, including sociodemographic factors like age, race, and education level, as well as clinical factors such as cancer stage and type of treatment. Numerical values and percentages are provided for each characteristic, indicating their distribution or frequency within the study population.

		Decline in physical well-being post-chemotherapy		Decline in physical well-being 6-months post-chemotherapy	
		Yes	No	Yes	No
Total participants:	*N* = 580	*N* = 310	*N* = 219	*N* = 178	*N* = 323
Age, years: mean [range]	53.4 [22-81]	53.7 [22-75]	52.5 [25-76]	54.2 [26-76]	52.7 [22-74]
Race: *N* (%)					
White	517 (89.1)	282 (91.0)	193 (88.1)	159 (89.3)	296 (91.6)
Non-white	63 (10.9)	28 (9.0)	26 (11.9)	16 (10.7)	27 (8.4)
Education: *N* (%)					
High school or below	142 (24.5)	74 (23.9)	52 (23.7)	130 (73.0)	251 (77.7)
Some college or above	438 (75.5)	236 (76.1)	167 (76.3)	48 (27.0)	72 (22.3)
Marital status: *N* (%)					
Married/long-term relationship	421 (72.6)	219 (70.6)	164 (74.9)	118 (66.3)	248 (76.8)
Other	159 (27.4)	91 (29.4)	55 (25.1)	60 (33.7)	75 (23.2)
Cancer stage: *N* (%)					
I	158 (27.3)	81 (26.1)	61 (27.9)	45 (25.3)	90 (27.9)
II	285 (49.1)	164 (52.9)	101 (46.1)	92 ()51.7	163(50.5)
III	108 (18.6)	50 (16.1)	47 (21.5)	37 ()20.8	51 (15.8)
Unknown	29 (5.0)	15 (4.8)	10 (4.6)	4 (2.2)	19 (5.9)
Type of treatment: *N* (%)					
Adjuvant	480 (82.8)	248 (80.0)	190 (86.8)	138 (77.5)	277 (85.8)
Neoadjuvant	100 (17.2)	62 (20.0)	29 (13.2)	40 (22.5)	46 (14.2)
Type of chemotherapy: *N* (%)					
Anthracycline	278 (47.9)	153 (49.4)	101 (46.1)	87 (48.9)	151 (46.7)
Non-anthracycline	303 (52.1)	157 (50.6)	118 (53.9)	91 (51.1)	172 (53.3)
Growth factor: *N* (%)					
Yes	471 (81.9)	266 (85.8)	188 (85.8)	152 (85.4)	277 (85.8)
No	109 (18.8)	44 (14.2)	31 (14.1)	26 (14.6)	46 (14.2)
Menopausal status: *N* (%)					
Pre	181 (31.2)	94 (30.3)	73 (33.3)	43 (24.1)	112 (34.7)
Peri	45 (7.8)	28 (9.0)	14 (6.4)	17 (9.6)	26 (8.1)
Post	303 (52.2)	158 (51.0)	115 (52.5)	99 (55.6)	158 (48.9)
Medically induced	51 (8.8)	30 (9.7)	17 (7.8)	19 (10.7)	27 (8.4)
BMI: mean [SD]	30.3 [7.4]	30.8 [7.3]	29.2 [7.5]	30.7 [7.3]	29.8 [7.5]
Time between labs and start of chemotherapy: mean [SD]	13.7 [35.2]	15.0 [39.5]	11.8 [28.9]	15.7 [34.5]	12.4 [37.3]
Cell Counts: Mean [SD]					
Total WBC (103 cells/µL)	7.5 [2.9]	7.4 [2.5]	7.7 [3.6]	7.3 [2.2]	7.7 [3.3]
Lymphocytes (103 cells/µL)	1.9 [1.0]	1.9 [0.8]	1.9 [1.2]	2.0 [0.8]	1.8 [1.1]
Monocytes (103 cells/µL)	0.5 [0.6]	0.5 [0.6]	0.5 [0.5]	0.5 [0.2]	0.6 [0.7]
Neutrophils (103 cells/µL)	4.7 [2.8]	4.6 [2.2]	4.9 [3.4]	4.4 [1.9]	4.9 [3.2]
Platelets (103 cells/µL)	266.6 [71.2]	267.4 [69.6]	266.9 [68.6]	262.4 [63.9]	272.2 [77.3]
NLR	3.0 [3.0]	2.8 [2.9]	3.2 [3.1]	2.5 [1.8]	3.3 [3.4]
LMR	5.7 [31.3]	7.1 [42.9]	4.3 [2.7]	4.9 [6.6]	6.7 [41.7]
PLR	159.5 [93.5]	157.6 [104.7]	163.6 [77.1]	146.8 [59.9]	169.1 [109.5]

Abbreviations: BMI, body mass index; LMR, lymphocyte to monocyte ratio; NLR, neutrophil to lymphocyte ratio; PLR, platelet to lymphocyte ratio platelet to lymphocyte ratio; WBC, white blood cell.

### Changes in physical well-being from pre-, to 1 month and 6 months post-chemotherapy

**Figure 1.**
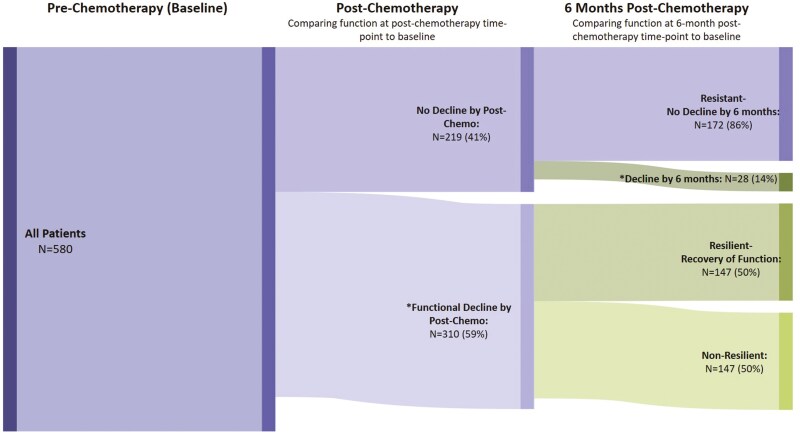
Flow diagram of changes in physical well-being, resilience and resistance in patients with breast cancer receiving chemotherapy from pre-chemotherapy to post-chemotherapy to 6 months post-chemotherapy: *Physical well-being decline: >one-point decrease on FACT-PWB from baseline **Resistant to decline: ≤one-point decrease on FACT-PWB from baseline to 6 months post-chemotherapy ***Non-resilience: those with physical well-being decline post-chemotherapy, and did not return to within 1 point on FACT-PWB of baseline. *Note: There were 51 patients with missing data to calculate physical well-being decline from the baseline to the post-chemotherapy time point and an additional 39 patients with missing data to calculate physical well-being decline and resilience by the 6-month post-chemotherapy time point.*

**Figure 2.**
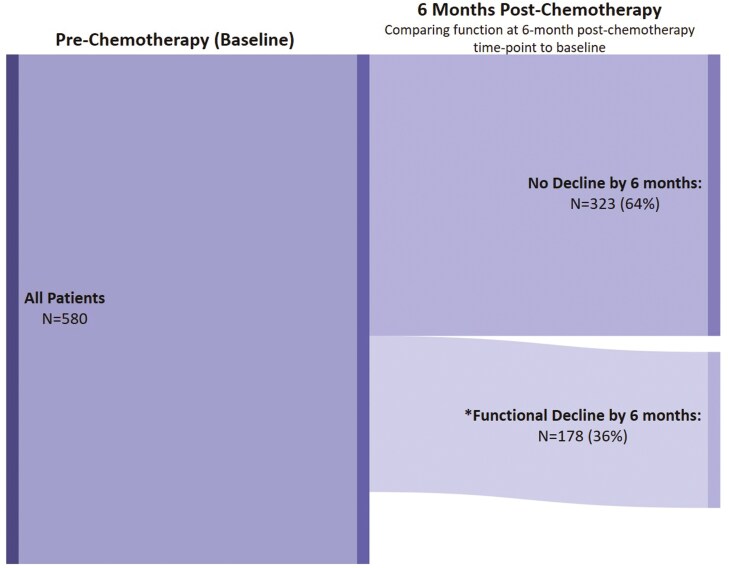
Flow diagram of changes in physical well-being in patients with breast cancer receiving chemotherapy from pre-chemotherapy to 6 months post-chemotherapy: *Physical well-being decline: more than one-point decrease on FACT-PWB from baseline. *Note*: There were 79 patients with missing data to calculate physical well-being decline from the baseline to the 6 months post-chemotherapy time point.

Patients’ mean FACT-PWB score was 22.2 at pre-chemotherapy (SD: 5.4), 19.4 at 1-month post-chemotherapy (SD: 5.8), and 22.5 at 6 months post-chemotherapy (SD: 4.8). Fifty-nine percent (310/529) of patients experienced physical well-being decline 1-month post-chemotherapy (Figure 1) and 36% (178/501) experienced physical well-being decline 6 months post-chemotherapy (Figure 2).

Of the patients with complete data from pre- to post-chemotherapy and post- to 6 months post chemotherapy, 147 (50%) were resilient and 147 (50%) were non-resilient. Furthermore, 172 (86%) were resistant to decline in physical well-being (Figure 1).

### Inflammation and physical well-being decline, from pre- to post-chemotherapy and from pre- to 6 months post-chemotherapy

There was no significant association between the inflammatory markers and physical well-being decline from pre-chemotherapy to post-chemotherapy (Table 2).

**Table 2.** Association of pre-chemotherapy cell counts and declines in physical well-being from pre- to post-chemotherapy and pre-to 6 months post-chemotherapy. Multivariable adjusted analyses were conducted to examine the association between pre-chemotherapy cell counts and declines in physical well-being from pre-chemotherapy to post-chemotherapy, and from pre-chemotherapy to 6 months post-chemotherapy. The results indicate that a low neutrophil-to-lymphocyte ratio was associated with a 1.78-fold increase in the odds of physical well-being decline from pre-chemotherapy to 6 months post-chemotherapy, while a low platelet-to-lymphocyte ratio was associated with a 1.66-fold increase in the odds of decline over the same period.

	Decline in physical well-being from pre- to post-chemotherapy (yes vs no decline)			Decline in physical well-being from pre- to 6 months post-chemotherapy (yes vs no decline)		Decline in physical well-being from pre- to post-chemotherapy (yes vs no decline)			Decline in physical well-being from pre- to 6 months post-chemotherapy (yes vs no decline)	
	Bivariate					Multivariable				
	N*	OR (95% CI)	*P* value	OR (95% CI)	*P* value	N*	OR (95% CI)	*P*value	OR (95% CI)	*P* value
WBC										
High	1030	1.00	0.51	1.00	0.77	965	1.00	0.44	1.00	0.76
Low		0.89 (0.63-1.26)		1.06 (0.73 - 1.53)			0.86 (0.58-1.27)		1.07 (0.71-1.61)	
Lymphocytes										
High	913	1.00	0.74	1.00	0.10	887	1.00	0.62	1.00	0.17
Low		1.06 (0.74-1.54)		0.72 (0.49-1.07)			1.11 (0.74-1.67)		0.74 (0.48-1.14)	
Monocytes										
High	917	1.00	0.17	1.00	0.37	891	1.00	0.18	1.00	0.51
Low		0.77 (0.53-1.12)		0.83 (0.56 - 1.24)			0.76 (0.50-1.14)		0.86 (0.56-1.34)	
Neutrophils										
High	909	1.00	0.21	1.00	0.88	883	1.00	0.37	1.00	0.73
Low		0.79 (0.55-1.14)		0.97 (0.66 - 1.44)			0.83 (0.55-1.25)		1.08 (0.70-1.66)	
Platelets										
High	963	1.00	0.41	1.00	0.70	961	1.00	0.45	1.00	0.75
Low		1.16 (0.81-1.66)		1.08 (0.74-1.58)			1.16 (0.79-1.70)		1.07 (0.71-1.61)	
LMR										
High	879	1.00	0.37	1.00	0.28	877	1.00	0.41	1.00	0.22
Low		1.19 (0.82-1.72)		0.80 (0.54-1.20)			1.18 (0.79-1.77)		0.77 (0.50-1.17)	
NLR										
High	883	1.00	0.80	**1.00**	**0.02**	881	1.00	0.61	**1.00**	**0.01**
Low		1.05 (0.72-1.52)		**1.62 (1.09 - 2.42)**			1.11 (0.75-1.65)		**1.78 (1.17-2.73)**	
PLR										
High	887	1.00	0.30	**1.00**	**0.01**	885	1.00	0.51	**1.00**	**0.02**
Low		1.22 (0.84-1.77)		**1.72 (1.15-2.57)**			1.14 (0.76-1.71)		**1.66 (1.07-2.57)**	

Models used were the generalized estimating equations models with logit link and an exchangeable structure. Multivariable models were adjusted for baseline physical well-being, age, race (white vs other), education (some college or above vs other), marital status (married/long term vs other), menopause status (peri vs pre, post vs pre, and medical induce vs pre), cancer stage (II vs I, III vs I, and IV vs I), body mass index, treatment type (adjuvant vs neo-adjuvant), growth factor (yes vs no), and time from lab blood draw to start of chemotherapy.

Inflammatory markers were cut at cohort median: high WBC: ≥7.00 × 10^3^ cells/µL, lymphocytes: ≥1.80 × 10^3^ cells/µL, monocytes: ≥0.50 × 10^3^ cells/µL, neutrophils: ≥4.10 × 10^3^ cells/µL, platelets: ≥257.5 × 10^3^ cells/µL, NLR: ≥2.27, LMR: ≥3.75, PLR: ≥145.27.

Values are bolded when the *P*-value of the association is *P*<0.05.

N*: number of observations used.

Abbreviations: LMR, lymphocyte to monocyte ratio; NLR, neutrophil to lymphocyte ratio; OR, odds ratio; PLR, platelet to lymphocyte ratio.

In bivariate analysis, low NLR was associated with 1.62-fold greater odds (95% CI = 1.09-2.42, *P* = .02) and low PLR was associated with 1.72-fold greater odds (95%CI = 1.15-2.57; *P* = .01) of having physical well-being decline from pre-chemotherapy to 6 months post-chemotherapy. In multivariable analysis low NLR was associated with 1.78-fold greater odds (95% CI = 1.17-2.73, *P* =.02) and low PLR was associated with 1.66-fold greater odds (95% CI = 1.07-2.57; *P* = .02) of having physical well-being decline from pre-chemotherapy to 6 months post-chemotherapy (Table 2). In sensitivity analyses, low lymphocytes were associated with 0.50 times lower odds (95% CI = 0.26-0.97; *P* = .04) of having physical well-being decline from pre-chemotherapy to 6 months post-chemotherapy. Low PLR remained significantly associated with 2.05-fold greater odds (95% CI = 1.10-3.83; *P* = .02) of having physical well-being decline from pre-chemotherapy to 6 months post-chemotherapy (Supplementary Table S1).

In bivariate analysis, of the patients with post-chemotherapy physical well-being decline, low lymphocyte counts were associated with 46% decreased odds (OR = 0.54; 95% CI = 0.33-0.89; *P* = .02) of being non-resilient by 6 months after completing chemotherapy. Low NLR was associated with 1.82-fold greater odds (95% CI = 1.10-3.01, *P* = .02) and low PLR was associated with 2.15-fold greater odds (95% CI = 1.30-3.56; *P* = .003) of being non-resilient by 6 months after completing chemotherapy. In multivariable analysis, low NLR was associated with 1.92-fold greater odds (95% CI = 1.11-3.33, *P* = .02) and low PLR was associated with 2.09-fold greater odds (95% CI = 1.91-3.67; *P* = .01) of being non-resilient by 6 months after completing chemotherapy (Table 3). In sensitivity analyses, we found that low PLR also remained significantly associated with 2.99-fold greater odds (OR = 2.99; 95% CI = 1.28-6.98; *P* = .01) of having physical well-being decline from pre-chemotherapy to 6months post-chemotherapy (Supplementary Table S1).

**Table 3.** In patients with post-chemotherapy decline in physical well-being: association of pre-chemotherapy cell counts and non-resilience at 6 months post-chemotherapy OR (95% CI). Multivariable adjusted analyses were conducted to examine the association between pre-chemotherapy cell counts and non-resilience at 6 months post-chemotherapy in patients who experienced a decline in physical well-being after chemotherapy. The results indicate that a low neutrophil-to-lymphocyte ratio was associated with a 1.92-fold increase in the odds of being non-resilient by 6 months after completing chemotherapy, while a low platelet-to-lymphocyte ratio was associated with a 2.09-fold increase in the odds of non-resilience over the same period.

	Non-resilience at 6 months post-chemotherapy (non-resilience vs resilience)					
Inflammatory markers	Bivariate			Multivariable		
	*N*	OR (95% CI)	*P* Value	*N*	OR (95% CI)	*P* Value
WBC						
High	294	1.00		277	1.00	
Low		0.89 (0.57-1.41)	.64		0.90 (0.53-1.54)	.70
Lymphocytes						
High	**257**	**1.00**		250	1.00	
Low		**0.54 (0.33-0.89)**	**.02**		0.57 (0.32-1.01)	.06
Monocytes						
High	258	1.00		251	1.00	
Low		0.73 (0.44-1.20)	.21		0.83 (0.47-1.45)	.51
Neutrophils						
High	257	1.00		250	1.00	
Low		0.99 (0.90-1.61)	.95		1.08 (0.62-1.87)	.79
Platelets						
High	277	1.00		277	1.00	
Low		1.17 (0.73-1.88)	.51		1.17 (0.69-1.97)	.57
LMR						
High	248	1.00		248	1.00	
Low		0.75 (0.45-1.23)	.25		0.74 (0.43-1.27)	.28
NLR						
High	**249**	**1.00**		**249**	**1.00**	
Low		**1.82 (1.10-3.01)**	**.02**		**1.92 (1.11-3.33)**	**.02**
PLR						
High	**249**	**1.00**		**249**	**1.00**	
Low		**2.15 (1.30-3.56)**	**.003**		**2.09 (1.91-3.67)**	**.01**

Multivariable models were adjusted for baseline physical well-being, age, race (white vs other), education (some college or above vs other), marital status (married/long term vs other), menopause status (peri vs pre, post vs pre, and medical induce vs pre), cancer stage (II vs I, III vs I, and IV vs I), body mass index, treatment type (adjuvant vs neo-adjuvant), growth factor (yes vs no), and time from lab blood draw to start of chemotherapy.

Inflammatory markers were cut at cohort median: high WBC : ≥ 7.00 × 10^3^ cells/µL, lymphocytes: ≥ 1.80 × 10^3^ cells/µL, monocytes: ≥ 0.50 × 10^3^ cells/µL, neutrophils: ≥ 4.10 × 10^3^ cells/µL, platelets: ≥ 257.5 × 10^3^ cells/µL, NLR: ≥ 2.27, LMR: ≥ 3.75, PLR: ≥ 145.27.

Values are bolded when the *P*-value of the association is *P*<0.05.

Abbreviations: LMR: lymphocyte to monocyte ratio; NLR: neutrophil to lymphocyte ratio; OR: odds ratio; PLR: platelet to lymphocyte ratio.

Finally, none of the inflammatory markers was significantly associated with resistance to decline in physical well-being (Table 4).

**Table 4.** In patients with no post-chemotherapy decline in physical well-being: association of pre-chemotherapy cell counts and non-resistance at 6 months post-chemotherapy OR (95% CI). Multivariable adjusted analyses were conducted to examine the association between pre-chemotherapy cell counts and non-resistance at the 6 months post-chemotherapy timepoint in patients who did not experience a decline in physical well-being after chemotherapy. The results show no association between any markers of inflammation and resistance to a decline in physical well-being.

	Non-resistance at 6 months post-chemotherapy (non-resistance vs resistance)					
Inflammatory markers	Bivariate			Multivariable		
	*N*	OR (95% CI)	*P* value	*N*	OR (95% CI)	P value
WBC						
High	200	1.00		183	1.00	
Low		2.39 (0.99-5.71)	0.05		2.52 (0.92-6.89)	0.07
Lymphocytes						
High	180	1.00		174	1.00	
Low		1.08 (0.47-2.50)	0.85		1.45 (0.56-3.77)	0.45
Monocytes						
High	181	1.00		175	1.00	
Low		1.71 (0.75-3.90)	0.20		1.35 (0.54-3.37)	0.52
Neutrophils						
High	178	1.00		172	1.00	
Low		1.35 (0.59-3.16)	0.47		1.36 (0.52-3.57)	0.53
Platelets						
High	184	1.00		183	1.00	
Low		0.79 (0.35-1.81)	0.58		0.50 (0.19-1.36)	0.17
LMR						
High	173	1.00		172	1.00	
Low		0.60 (0.25-1.4)	0.25		0.81 (0.31-2.12)	0.66
NLR						
High	173	1.00		172	1.00	
Low		1.54 (0.66-3.58)	0.31		1.72 (0.67-4.42)	0.26
PLR						
High	175	1.00		174	1.00	
Low		0.99 (0.43-2.29)	0.99		0.72 (0.27-1.92)	0.51

Multivariable models were adjusted for baseline physical well-being, age, race (white vs other), education (some college or above vs other), marital status (married/long term vs other), menopause status (peri vs pre, post vs pre, and medical induce vs pre), cancer stage (II vs I, III vs I, and IV vs I), body mass index, treatment type (adjuvant vs neo-adjuvant), growth factor (yes vs no), and time from lab blood draw to start of chemotherapy.

Inflammatory markers were cut at cohort median: High WBC: ≥ 7.00 × 10^3^ cells/µL, llymphocytes: ≥ 1.80 × 10^3^ cells/µL, monocytes: ≥ 0.50 × 10^3^ cells/µL, neutrophils: ≥ 4.10 × 10^3^ cells/µL, platelets: ≥ 257.5 × 10^3^ cells/µL, NLR: ≥ 2.27, LMR: ≥ 3.75, PLR: ≥ 145.27.

Values are bolded when the *P*-value of the association is *P*<0.05.

Abbreviations: LMR, lymphocyte to monocyte ratio; NLR, neutrophil to lymphocyte ratio; R, odds ratio; PLR, platelet to lymphocyte ratio.

## Discussion

In this prospective cohort of patients with breast cancer who were receiving chemotherapy, we found that a large proportion of patients had chemotherapy-induced decline in physical well-being. We also showed that in patients mean age = 53.4 years, 59% of patients experienced decline in physical well-being 1-month after completing chemotherapy and 36% experienced decline in physical well-being 6 months after the completion of chemotherapy. Furthermore, we found that of the patients who experienced decline in physical well-being 1-month after completing chemotherapy, 50% were resilient, indicating they resumed their level of physical well-being before they started chemotherapy. We went on to demonstrate that patients with a low level of NLR and PLR were twice as likely to have a decline in their physical well-being from the start to end of chemotherapy and to be non-resilient by 6 months after completing chemotherapy, after controlling for potential confounders. To the best of our knowledge, this is the first study to specifically investigate the role of these inflammatory markers using laboratory data on physical well-being in patients with breast cancer who were receiving chemotherapy.

In the context of cancer, NLR and PLR are typically investigated as markers of systemic inflammatory responses during the development and progression of cancer. Inflammatory markers, specifically NLR and PLR may stratify patients based on their risk of chemotherapy-induced decline in physical well-being prior to the start of treatment and are routinely used to guide clinicians during the diagnosis and treatment of patients. NLR and PLR have been widely studied as markers of systemic inflammation and the majority of oncology studies have shown that elevated levels of NLR and PLR are predictive and prognostic markers of survival and frailty in patients with cancer.^29-32^ Unexpectedly our findings were contrary to these common findings. We found an inverse relationship between these inflammatory markers and physical well-being decline, with lower pre-chemotherapy levels of NLR and PLR associated with physical well-being decline and non-resilience 6 months after completing chemotherapy. Only one study to date in patients with breast cancer has demonstrated a similar predictive trend of NLR with outcomes. Grassadonia et al found that in women with breast cancer, low pre-treatment NLR (< 2.12) was associated with increased risk of cancer recurrence and mortality.^33^ In other disease settings, however, low NLR and PLR has been shown to be associated with negative outcomes.^34-36^ The unexpected inverse correlation observed between inflammatory markers and the decline in physical well-being may be attributed to the varying interactions between these inflammation markers and the distinct domains measured by the FACT-PWB; eg, nausea, fatigue, and pain. Subsequent research endeavors should aim to elucidate the complexities inherent in this association. These studies truly highlight the complexity of determining the precise role of these inflammatory markers as predictive and prognostic markers and suggest that there is a non-linear relationship between inflammation and clinical outcomes in patients with cancer which warrants further investigation.

An elevated NLR is due to a relative neutrophilia and lymphocytopenia that occurs as part of the systemic inflammatory response to tumorigenesis, while an elevated PLR has been proposed to be due to the pro-inflammatory properties of platelets.^37-40^ However, it is important to note that neutrophils, lymphocytes, and platelets are heterogeneous cell populations (eg, lymphocytes consist of natural killer cells, T-cells, and B-cells) each with distinct roles.^41-44^ In our study we are unable to determine the precise role of specific immune subsets on chemotherapy-induced physical well-being decline. Future studies, using immunoprofiling techniques, (eg, flow cytometry) are needed elucidate the specific roles of the individual inflammatory markers and shed light on the potential non-linear relationship between inflammation and clinical outcomes in patients with cancer.

Chemotherapy-induced decline in physical well-being is a critical impairment for patients with breast cancer and increases the risk of loss of independence, hospitalization, and mortality.^45-48^ The results from our study suggest that more than half of patients with breast cancer who are receiving chemotherapy may benefit from targeted interventions to prevent and/or treat chemotherapy-induced physical well-being decline and promote resilience.

Given that patients with cancer frequently experience symptoms such as pain, nausea, and fatigue, which are closely linked to reduced physical function and diminished quality of life, we employed the FACT-PWB scale to assess changes in physical well-being and resilience within this cohort.^4,5^ We recognize that, unlike previous studies that have evaluated resilience through specific measures centered on functional quality of life, our study did not utilize a specific measure of physical function and instead we used the FACT-PWB which encompasses aspects of function and symptoms. Despite this difference, we saw analogous patterns in decline as the Hurria et al. study which used the European Organization for Research and Treatment of Cancer (EORTC) physical function subscale as well as symptom items. In Hurria’s study of older adults with breast cancer, 52% had functional decline from pre- to post-chemotherapy along with 30% having worsening in symptoms from start of treatment to 12 months post-chemotherapy with 53% resilient.^49^ In our cohort, including younger women with breast cancer, we demonstrated that 59% of patients experienced decline in physical well-being 1 month after completing chemotherapy and 36% experienced decline in physical well-being 6 months after the completion of chemotherapy and 50% were resilient to decline in physical well-being. Altogether these results demonstrate that chemotherapy-induced functional decline and decline in physical well-being are prevalent in older and younger women with breast cancer. Comprehending the mechanisms underlying these interrelated concepts is essential for informing the development of interventions that address alterations in both physical functioning and physical well-being for women with breast cancer receiving chemotherapy.

Despite a growing awareness of key survivorship issues faced by patients with breast cancer including increased risk of multimorbidity, frailty, and physical well-being decline earlier than would be expected in the general population, there is a paucity of interventions to prevent, delay, or treat these short- and long-term adverse effects.^50^ Our work showing the adverse effect of low NLR and PLR on chemotherapy-induced decline in physical well-being is consistent with research demonstrating that inflammation may be a key targetable biological pathway that could be leveraged to prevent and/or treat chemotherapy-induced decline in physical well-being and promote resilience.^51^ Behavioral and nutritional interventions known to modulate inflammatory pathways could be potential interventions for chemotherapy-induced changes to physical well-being, once we are able to unravel essential complexities of immune function in this setting.^52^ For example, exercise is considered a safe and cost-effective method of improving clinical outcomes and preserving physical well-being in cancer survivors through its anti-inflammatory effects.^53^ In patients with breast cancer, exercise has been shown to reduce inflammation through the increased production of circulating anti-inflammatory cytokine-producing T-cells.^54^ Diet and dietary supplements have also been shown to play a significant role in altering the serum concentration of a variety of systemic markers of inflammation.^55-57^

This study had several strengths. First, while many studies in patients with cancer evaluated the predictive role of inflammatory markers on survival, our study examined its association with changes in physical well-being after cancer treatment. This is important because limitations in physical well-being and quality of life as a side effect of chemotherapy is one of the key survivorship issues for patients with breast cancer and particularly older patients due to its negative effect on independent living and quality of life. Second, this study used available data from a prospective longitudinal cohort study that enrolled participants from multiple community oncology sites within the US, making our findings generalizable to patients seen in oncology clinics within community settings that traditionally treat the majority of patients with cancer. Third, this was a large (*n* = 580) prospective longitudinal study with physical well-being measures obtained at multiple time points, allowing us to observe the predictive potential of pre-treatment cellular markers of inflammation with chemotherapy-induced changes to physical well-being.

Our study also has limitations. Due to the secondary nature of our analysis, our study was confined to the pre-existing dataset. Consequently, we utilized the physical well-being domain in the quality-of-life measure available in our dataset to assess chemotherapy-induced non-resilience as a measure of patients’ inability to return to pre-chemotherapy levels of well-being. It is important to note that this approach of using the FACT-PWB deviates from conventional methods of assessing resilience. Leveraging the established clinical significance of a 1-point change specifically in the FACT-PWB domain,^23^ we applied this criterion to evaluate resilience in physical well-being. Future research should seek to establish clinically meaningful differences in FACT-PWB subdomains for capturing resilience in physical well-being. Additionally, in this study we only assessed physical well-being up to 6 months after the completion of chemotherapy. We know that chemotherapy-induced physical well-being decline is a dynamic process and can last for years after treatment. Thus, it is unclear the long-term predictive potential of cellular markers of inflammation on non-resilience. Future work should assess whether, pre-treatment cellular markers of systemic inflammation are associated with physical well-being decline and non-resilience years after the completion of treatment. We also know that how inflammatory markers change over the course of treatment is predictive of outcomes such as frailty.^21,51^ While the intent of this current study was to investigate the association of pre-treatment inflammatory markers, future work should also assess whether changes in these inflammatory markers are associated with chemotherapy-induced physical well-being decline and non-resilience. Furthermore, physical well-being was obtained through self-report using a 7-item scale, rather than an objective measure of physical function like the Short Physical Performance Battery (SPPB) test^58^ and could be subject to biases. However, there is growing consensus that how people subjectively feel about their physical well-being status is as important as objective findings of physical function. Imputing missing biomarkers of inflammation values may not be appropriate or reliable, especially if the missingness is related to the biomarker levels themselves (ie, missing not at random). Imputing such values could potentially introduce bias in the estimates. In terms of sample size considerations, despite the missing data, our complete-case sample sizes (the minimum sample sizes: 878 for the analyses of the associations between baseline levels of inflammatory markers at pre-chemotherapy and physical well-being declines post-chemotherapy and 6 months post-chemotherapy, 248 with decline post-chemotherapy used for the analysis of resilience at 6 months post-chemotherapy, and 172 without decline post-chemotherapy used for the analysis of resistance at 6 months post-chemotherapy) were reasonably large, providing adequate statistical power for the multivariable GEE models and the multivariable logistic regression models. While we acknowledge the potential limitations of complete-case analysis, such as a loss of precision, we believe that using complete-case data is a more transparent and reliable approach in this context, given the nature of the predictors. This approach avoids the additional assumptions and potential biases introduced by imputing biomarker values.

## Conclusion

This study underscores the link between inflammation and chemotherapy-induced decline in physical well-being and sets the groundwork for future studies and development of interventions to circumvent and/or treat chemotherapy-induced physical well-being decline and promote resilience in patients with breast cancer. Additional research is needed to determine the clinical utility of inflammatory biomarkers in identifying patients at most risk of decline. Given the complexity of the components and functions of the immune system, the precise role of cellular markers of inflammation on chemotherapy-induced physical well-being decline, should be evaluated using immunoprofiling techniques such as flow-cytometry, multiplex immunofluorescence, genomics, and/or proteomics. In patients with breast cancer receiving chemotherapy, low levels of NLR and PLR are associated with physical well-being decline and non-resilience up to 6 months after completing treatment. These profiles may help clinicians identify the patients who are at increased risk of physical well-being decline and non-resilience and inform treatment discussions. Additional research is needed to determine the clinical utility of inflammatory biomarkers in identifying patients at most risk of decline before the start of chemotherapy.

## Supplementary Material

oyae212_suppl_Supplementary_Table_S1

## Data Availability

The data described in the article may be made available upon application and approval.

## Prior presentation

A portion of this work has been presented at the Annual American Society of Clinical Oncology conference, May 29-31, 2020.

## Trial registration

ClinicalTrials.gov Identifier: NCT01382082. Registered June 27, 2011.
