# Correction to: Systemic inflammation and changes in physical well-being in patients with breast cancer: a longitudinal study in community oncology settings

**DOI:** 10.1093/oncolo/oyae278

**Published:** 2024-10-01

Nikesha Gilmore, Yue Li, Christopher L Seplaki, Michael Sohn, Ying Wang, Chin-Shang Li, Kah Poh Loh, Po-Ju Lin, Amber Kleckner, Mostafa Mohamed, Paula Vertino, Luke Peppone, Karen Mustian, Sindhuja Kadambi, Steven W Corso, Benjamin Esparaz, Jeffrey K Giguere, Supriya Mohile, Michelle C Janelsins, Systemic inflammation and changes in physical well-being in patients with breast cancer: a longitudinal study in community oncology settings, *The Oncologist*, 2024;, oyae212, https://doi.org/10.1093/oncolo/oyae212

The fifth author’s name has been corrected.

