# Supplementary material for: Systemic inflammation and changes in physical well-being in patients with breast cancer: a longitudinal study in community oncology settings
Source: Oncologist. 2024 Aug 23;30(2):oyae212. doi: 10.1093/oncolo/oyae212 (PMC11881059; doi:10.1093/oncolo/oyae212)
Supplement: oyae212_suppl_Supplementary_Table_S1 [file oyae212_suppl_supplementary_table_s1.docx]

Supplemental table 1 Association of Pre-Chemotherapy Cell Counts and Physical function decline post chemotherapy

| **Inflammatory Markers**  **(Quartile 1 vs. Quartile 4)** | **N*** | **OR (95% CI)** | **P Value** | **OR (95% CI)** | **P Value** |
| --- | --- | --- | --- | --- | --- |
|  |  | **Decline in Physical Well-being from Pre- to Post-Chemotherapy (Yes vs No Decline)** | | **Decline in Physical Well-being from Pre- to Six months Post-Chemotherapy (Yes vs No Decline)** | |
| WBC | 961 | 0.61 (0.35 - 1.06) | 0.08 | 0.84 (0.48 - 1.50) | 0.56 |
| Lymphocytes | 887 | 0.61 (0.34 - 1.11) | 0.10 | **0.50 (0.26 – 0.97)** | **0.04** |
| Monocytes | 891 | 0.71 (0.40 - 1.24) | 0.23 | 0.77 (0.42 - 1.42) | 0.41 |
| Neutrophils | 883 | 0.94 (0.53 - 1.68) | 0.84 | 0.92 (0.51 - 1.69) | 0.80 |
| Platelets | 961 | 1.05 (0.61 – 1.82) | 0.86 | 1.47 (0.82 - 2.63) | 0.20 |
| LMR | 877 | 1.26 (0.72 - 2.22) | 0.42 | 0.72 (0.40 - 1.31) | 0.28 |
| NLR | 881 | 1.02 (0.57 – 1.81) | 0.96 | 1.49 (0.81 - 2.74) | 0.20 |
| PLR | 885 | 1.27 (0.71 - 2.30) | 0.42 | **2.21 (1.16 – 4.19)** | **0.02** |
|  |  | **OR (95% CI)** | | **P Value** | |
|  |  | **Non-Resilience at Six months Post-Chemotherapy (Non-Resilience vs Resilience)** | | | |
| WBC | 277 | 0.89 (0.43 - 1.85) | | 0.75 | |
| Lymphocytes | 250 | 0.54 (0.23 - 1.26) | | 0.15 | |
| Monocytes | 251 | 0.93 (0.44 - 1.96) | | 0.85 | |
| Neutrophils | 250 | 0.83 (0.38 - 1.80) | | 0.63 | |
| Platelets | 277 | 1.93 (0.91 - 4.08) | | 0.08 | |
| LMR | 248 | 0.69 (0.33 - 1.47) | | 0.34 | |
| NLR | 249 | 1.21 (0.54 - 2.69) | | 0.64 | |
| PLR | 249 | **2.99 (1.28 - 6.98)** | | **0.01** | |
|  |  | **Non-Resistance at Six months Post-Chemotherapy (Non-Resistance vs Resistance)** | | | |
| WBC | 183 | 1.34 (0.32 - 5.67) | | 0.69 | |
| Lymphocytes | 174 | 0.85 (0.20 - 3.58) | | 0.83 | |
| Monocytes | 175 | 0.61 (0.15 - 2.40) | | 0.48 | |
| Neutrophils | 172 | 1.07 (0.29 - 3.91) | | 0.92 | |
| Platelets | 183 | 0.29 (0.07 - 1.22) | | 0.09 | |
| LMR | 172 | 1.07 (0.27 - 4.22) | | 0.92 | |
| NLR | 172 | 2.03 (0.47 - 8.76) | | 0.34 | |
| PLR | 174 | 0.49 (0.13 - 1.92) | | 0.31 | |
| We grouped participants by quartiles of inflammatory makers. We present odds ratio of change in physical function for the lowest quartile compared with the highest quartile (Quartile 1 vs. Quartile 4). Each multivariable models was individually adjusted for baseline function, age, race (white vs other), education (some college or above vs other), marital status (married/long term vs other), menopause Status (peri vs pre, post vs pre, and medical induce vs pre), cancer stage (II vs I, III vs I, and IV vs I), body mass index, treatment type (adjuvant vs neo-adjuvant), growth factor (yes vs no), and time from lab blood draw to start of chemotherapy.  Abbreviations: OR: Odds Ratio; CI: Confidence Interval; NLR: Neutrophil to Lymphocyte ratio; LMR: Lymphocyte to Monocyte ratio; PLR: Platelet to Lymphocyte ratio.  N*: Number of observations used | | | | | |
